# Trust and world view in shared decision making with indigenous patients: A realist synthesis

**DOI:** 10.1111/jep.13307

**Published:** 2019-11-21

**Authors:** Gary Groot, Tamara Waldron, Leonzo Barreno, David Cochran, Tracey Carr

**Affiliations:** ^1^ Department of Community Health and Epidemiology University of Saskatchewan Saskatoon SK Canada

**Keywords:** indigenous, patients, realist review, shared decision making, trust, world view

## Abstract

**Introduction:**

How shared decision making (SDM) works with indigenous patient values and preferences is not well understood. Colonization has affected indigenous peoples' levels of trust with institutions, and their world view tends to be distinct from that of nonindigenous people. Building on a programme theory for SDM, the present research aims to refine the original programme theory to understand how the mechanisms of trust and world view might work differently for indigenous patients.

**Design:**

We used a six‐step iterative process for realist synthesis: preliminary programme theory development, search strategy development, selection and appraisal of literature, data extraction, data analysis and synthesis, and formation of a revised programme theory.

**Data Sources:**

Searches were through Medline, CINAHL, and the University of Saskatchewan iPortal for grey literature. Medline and CINAHL searches included the University of Alberta Canada‐wide indigenous peoples search filters.

**Data synthesis:**

Following screening 731 references, 90 documents were included for data extraction (53 peer reviewed and 37 grey literature). Documents from countries with similar colonization experiences were included.

**Results:**

A total of 518 context‐mechanism‐outcome (CMO) configurations were identified and synthesized into 21 CMOs for a revised programme theory. Demographics, indigenous world view, system and institutional support, language barriers, and the macro‐context of discrimination and historical abuse provided the main contexts for the programme theory. These inspired mechanisms of reciprocal respect, perception of world view acceptance, and culturally appropriate knowledge translation. In turn, these mechanisms influenced the level of trust and anxiety experienced by indigenous patients. Trust and anxiety were both mechanisms and intermediate outcomes and determined the level of engagement in SDM.

**Conclusion:**

This realist synthesis provides clinicians and policymakers a deeper understanding of the complex configurations that influence indigenous patient engagement in SDM and offers possible avenues for improvement.

## 1 INTRODUCTION

Early encounters between Europeans and indigenous peoples in what is now Canada resulted in peace and friendship treaties, commercial agreements, military alliances, and the numbered treaties.^1^ In the late 1800s, the implementation of these mutually beneficial agreements began to change. Indian Residential Schools and government regulations of indigenous identity (eg, the Indian Act) contributed to indigenous institutional and cultural distrust of government policies.^2^, ^3^, ^4^, ^5^ Multilayered discrimination has been linked to current negative health outcomes^6^, ^7^ and disparities^8^, ^9^, ^10^, ^11^ in health services to indigenous populations. Power imbalances and a shortage of health‐care provider (HCP) education regarding indigenous world views are believed to add to these inequalities.^12^, ^13^ For a definition of indigenous peoples and other key terms used in our research, see Table 1.

**Table 1:** Definitions of the terminology

Terminology	Definition
Indigenous peoples	In Canada: term that collectively refers to First Nations, Métis, and Inuit^14^
	Globally: According to the World Health Organization, they are distinct cultural groups that reside within or have relationships to land, specifically land that their ancestors occupied before modern states and borders were defined. They “maintain cultural and social identities, and social, economic, cultural and political institutions, separate from the mainstream or dominant society or culture.”^15^
Shared decision making	Process in which both the patient and physician contribute to the medical decision‐making process (eg, tests, treatments, and care plans)^16^
Context‐mechanism‐outcome configuration	Model that involves identifying the context, mechanism, and outcome pattern configuration to determine what works for whom and in what circumstances (Context + Mechanism = Outcome)^17^
Context	Something that can impact or even block a Mechanism. The Context may be the type of intervention, the type of population, or a broader contextual “backdrop” within which the programme/intervention operates^17^
Mechanism	The generative force that results in an Outcome. It can be manifested as reasoning and/or response to the resources or capabilities offered by or embedded in a programme/intervention^17^
Outcome	What happened as a result of the Context and Mechanism (intentional or unintentional)^17^
Grey literature	Documents that are not published through traditional sources (eg, academic journals) but that may contain information relevant to a review. Examples of grey literature documents include clinical trials, theses, censes, and government reports^18^

Shared decision making (SDM) emphasizes equalizing power between patients and HCPs to create more equitable health care.^16^ However, limited research exists to examine if and how SDM works with indigenous patient values and preferences.^19^, ^20^, ^21^ For instance, a systematic review identified one study that examined SDM with indigenous patients where a generic model was applied without cultural adaptation.^22^ When culturally adapted SDM tools have been implemented, 20 indigenous women felt the tools were from a Westernized lens and lacked incorporation of indigenous beliefs.^19^ This suggests that further work is required to implement an SDM model that meets the needs of indigenous patients.

In an earlier realist synthesis, we explored the following: “In which situations, how, why, and for whom does SDM between patients and HCPs contribute to improved engagement in the shared decision‐making process?”^23^ We outlined eight key mechanisms that impact engagement in the SDM process.^24^ How we used this initial programme theory as the basis of the present study to confirm, refine, or refute the findings when applied to indigenous patients is discussed in Section 2.

On the basis of qualitative interviews with indigenous patients with cancer, we recognized that indigenous patients often hold different world views from those of HCPs and the health‐care system.^25^ To understand how trust and perceptions of world view work with indigenous patients to impact SDM, our research question was as follows: “in a healthcare consultation involving Indigenous patients, for whom, why and in what situations do trust and perceptions of world view influence patient engagement to achieve SDM?”

## 2 METHODS

### 2.1 Methodology: Realist synthesis

Realist syntheses aim to explain how, why, for whom, and in which circumstances an intervention succeeds or fails.^26^, ^27^, ^28^ To achieve this, contexts (C), mechanisms (M), and outcomes (O) are extracted from existing literature and configured into explanations of why, how, and for whom an outcome occurs (ie, if processes appear [M] in the right conditions [C], then an outcome [O] will result). 29 The benefit of realist syntheses is that they are theory based, which enables researchers to develop or test a programme theory.^28^, ^29^, ^30^

Realist syntheses extend beyond a traditional literature review by developing programme theories composed of testable hypotheses.^31^ Testable hypotheses are represented as CMO configurations as a middle‐range theory,^27^ which is a level of abstraction that assists in explaining regularities in social behaviour.^24^, ^29^ Unlike literature reviews, realist syntheses present the opportunity to both build and refine theory. Our intention is to refine our SDM programme theory with a view that is potentially useful for HCPs and their indigenous patients as they navigate clinical encounters; therefore, realist synthesis was the most appropriate method. The aim of this research was to better understand how the key mechanisms of trust and world view identified in our prior realist synthesis of SDM might work differently with indigenous patients and if so why, for whom, and in what circumstances.

We conducted a six‐step iterative process for realist syntheses^24^, ^32^: preliminary programme theory development, search strategy development, selection and appraisal of literature in accordance with realist methodology,^31^, ^33^ data extraction, data analysis and synthesis, and formation of a revised programme theory. This follows Pawson's explanation of how to conduct a realist synthesis and aligns with RAMESES guidelines.^30^, ^31^, ^33^

**Figure 1:**
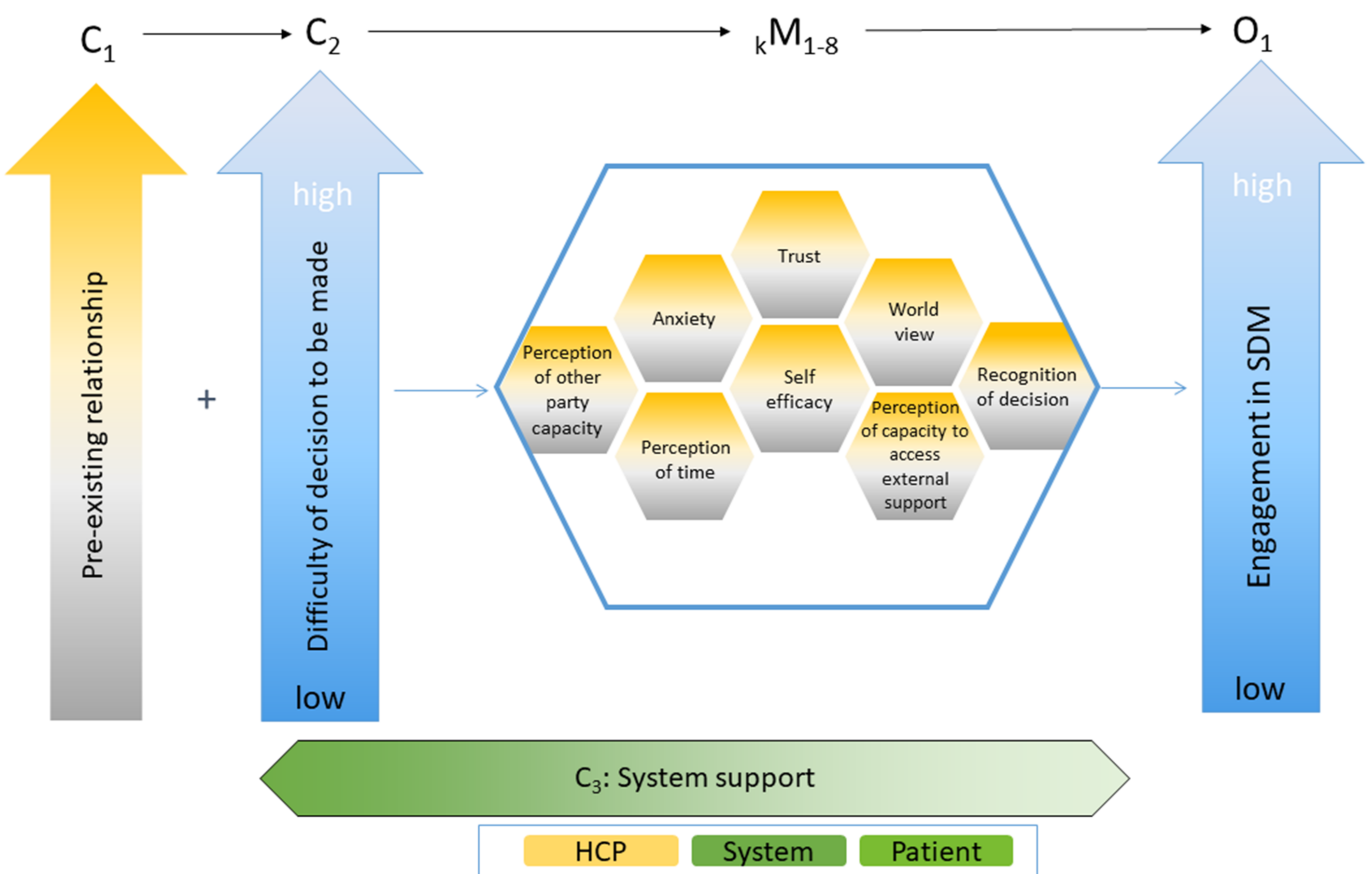
Original programme theory of shared decision making

The first step, the preliminary programme theory development, established the rationale for this study.^23^ Our first project developed an SDM programme theory by exploring the following: “In which situations, how, why, and for whom does SDM between patients and HCPs contribute to improved engagement in the shared decision‐making process?”^24^ The result was eight key mechanisms that impact engagement in SDM (see Figure 1).^23^ Shared decision making, in this sense, is considered an intervention that results in positive outcomes for the patient, the practitioner, and the health‐care system.^18^, ^34^, ^35^ Our initial programme theory served as the theoretical base in which we confirmed, refined, or refuted the findings when applied to an indigenous context.^23^ Indigenous peoples have been exposed to colonial forces that have affected their levels of trust with institutions,^3^, ^4^, ^5^ and at the same time, they have a world view that tends to be distinct from that of nonindigenous people.^36^, ^37^, ^38^ Therefore, we chose to focus on the mechanisms of trust and world view because of the unique characteristics of indigenous patients.

### 2.2 Systematic search strategy

Searches were through Medline, and CINAHL, and the University of Saskatchewan iPortal, which we used as our primary database for grey literature. Medline and CINAHL searches included the University of Alberta Canada‐wide indigenous peoples search filters. In addition to these filters, search terms for all databases included the following: “Indians, north american/,” “aboriginal*.mp.,” “native*.mp.,” “Health services, indigenous/,” “exp American Native Continental Ancestry Group/or Oceanic Ancestry Group/,” “indigenou*.mp.” “choice behavior/or choice behavio*.mp.,” “decision making/,” “decision*.mp.,” “(choic* or prefence*).mp.,” “culture/or culture.mp. or world view.mp. or world view.mp.,” and “trust/or trust.mp.” iPortal search criteria included “Decision Making,” “trust,” “world view,” and “worldview.”

### 2.3 Search process

Iterative screening was completed by two team members. A first round of title and abstract screening was conducted, followed by full‐text screening. All grey literature articles underwent full‐text screening. Inclusion criteria included the following: indigenous focus, English language, origin in Canada, United States, Australia, New Zealand, and patient/community member view. These countries were selected for inclusion because they share a similar history of colonization and segregation of indigenous populations. Exclusion criteria were as follows: HCP view only, government view only, programme description, policy, book review, and population under 18 years. Reviewers relied on realist relevance and rigour criteria as outlined by Pawson to assess the applicability and methodological appropriateness, credibility, trustworthiness, and methodologically sound in relation to the research design.^26^ Relevance was determined by whether the source could contribute to refinement of the theory, while rigour incorporated the credibility and trustworthiness of the source. All screening and extraction of peer‐reviewed documents were conducted through Covidence, while grey literature analysis was conducted in NVivo 11 and Microsoft Excel.

Two authors independently read each article and extracted CMOs. The process of identifying excerpts was guided by our main research questions with the intent of identifying key elements of trust and world view. The extraction template included identification of the contexts, mechanisms, and outcomes; a brief description of the paper; and bibliographic information. The CMOs were entered into either Covidence (peer‐reviewed sources) or NVivo 11 and Microsoft Excel (grey literature) for indexing. We then linked CMOs to a preliminary analytic framework that was iteratively reviewed and adjusted.^39^ The preliminary framework was based on our initial programme theory where CMOs were analysed according to how they confirmed, refined, or refuted the theory. The disconfirming data, in particular, illustrated the distinct characteristics of this group of patients. The process of analysis and synthesis is described in greater detail in Section 3.

## 3 RESULTS

### 3.1 Document characteristics

**Figure 2:**
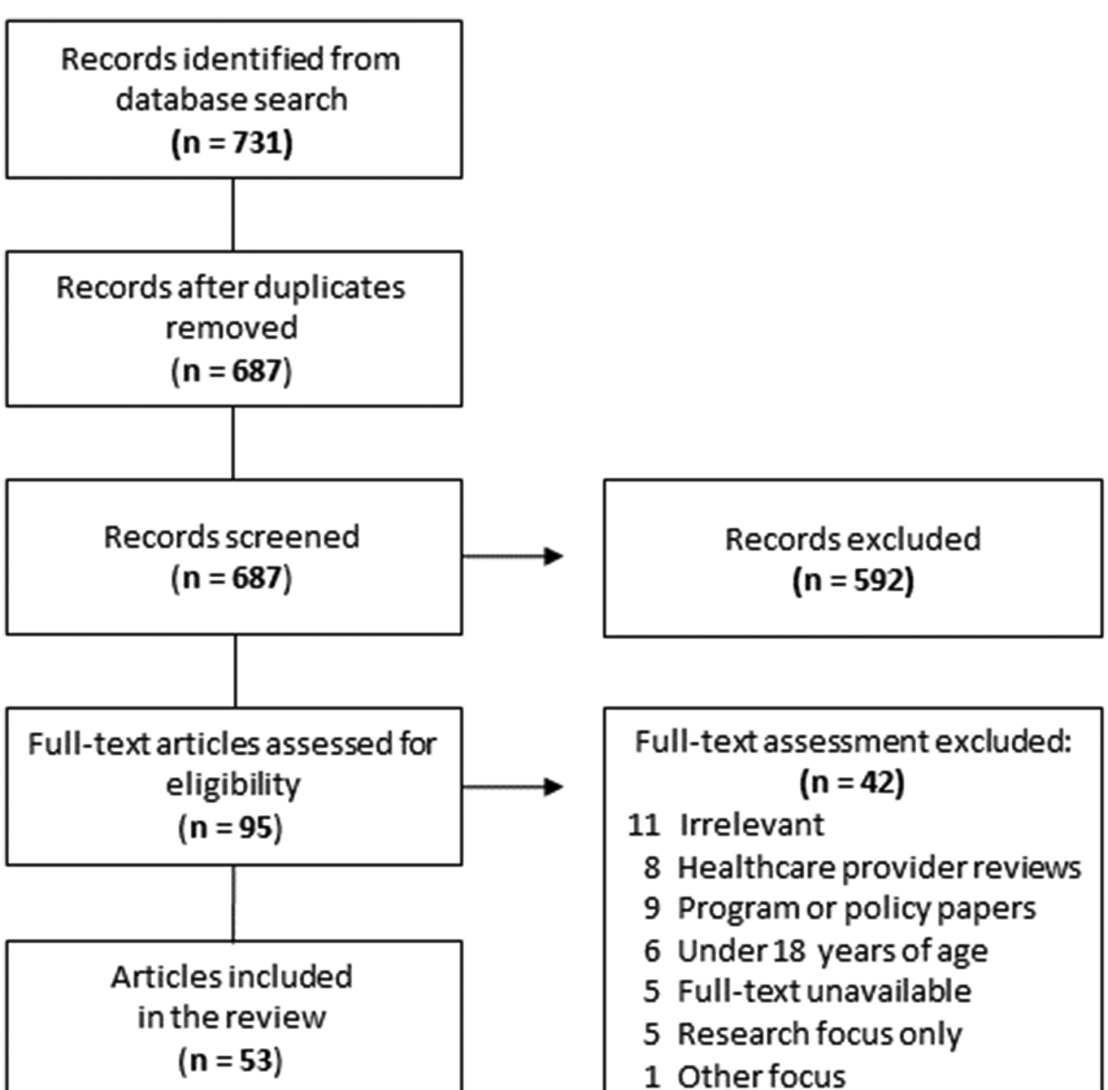
Flow diagram illustrating the screening process of peer‐reviewed literature

The search resulted in 731 references (see Figure 2). After de‐duplication, the result was 687 original sources; 95 articles underwent full‐text screening, and 42 were excluded. Following complete screening, a total of 90 documents were included for data extraction (including 53 peer reviewed and 37 from grey literature). The peer‐reviewed literature comprised 26 qualitative studies, 10 review articles, seven editorials, five mixed methods, and five quantitative studies. Grey literature documents included transcripts of the Canadian Royal Commission of Aboriginal Peoples, newspaper articles, report disseminations, and thesis documents.

### 3.2 Analysis and synthesis

Data, in the form of CMOs, were extracted from all screened sources. A total of 518 CMO configurations were identified. These CMOs included a mechanism of trust and/or world view and intermediate outcomes. The CMOs were then synthesized, based on demi‐regularities (ie, patterns).^24^ We iteratively classified the demi‐regularities into contexts and mechanisms on the basis of a process by Pawson and colleagues.^24^ Throughout the synthesis process, demi‐regularities within the data were refined based on barriers or enablers to SDM among indigenous patients.^39^ When the final synthesis was complete, 21 key CMOs had been identified. The next step in analysis was to use the initial programme theory as the template to contrast with our findings. We determined how well each of these context and mechanisms fit with the existing theory and how many were unique to the data from indigenous patients.

While some of the mechanisms within the existing theory still applied (ie, trust, world view, and anxiety), others acted as contexts for indigenous patients. For example, the mechanism “perception of time” was more appropriate as a context within indigenous world view. In addition, “perception of other party capacity” was refined as part of the mechanism “reciprocal respect” in the revised programme theory. In the final analysis, the existing theory was significantly refined to reflect indigenous patients' decision‐making experiences.

As a result, a macro‐context was identified as cultural discrimination and historical abuse that overlays the entire medical consultation. Four new contexts were developed: demographics (C_1_), indigenous world view (C_2_), system and institutional support (C_3_), and language barriers (C_4_). Reciprocal respect (M_1_), culturally perception of world view acceptance (M_2_), appropriate knowledge translation for HCP (M_3_), trust (M_4_), and anxiety (M_5_) were mechanisms in the revised programme theory. During our analysis of the CMO configurations, we determined that trust and anxiety could act as both mechanisms and intermediate outcomes. Components of both contexts and mechanisms related to the revised programme theory he configurations of C, M, _I_O (intermediate O) and/or _F_O are compiled in Table 2.

**Table 2:** CMO configurations in revised programme theory

Category	Attribute	Detailed CMOs
Demographics *Context set* (C_1_)	Gender	1. Men (C) + traditional indigenous world view on decision making (M) ➔ more likely to engage in SDM (O)
		2. Women (C) + traditional indigenous world view on decision making (M) ➔ less likely to engage in SDM (O)
	Age	1. Younger individual (C_1_) who has experienced trauma from historical colonization (_m_C) + lower alignment with an indigenous world view (M) ➔ higher willingness to engage in the biomedical health system (O)
	Location	1. On‐reserve home location (C) + *greater influence of community values* (M) ➔ higher alignment with an indigenous world view (O)
		2. Off‐reserve home location (C) + *lower influence of community values* (M) ➔ lower alignment with an indigenous world view (O)
Indigenous world view *Context set* (C_2_)	Perception of time	1. Perception of time (C) + (M) ➔ unlikely to seek Western medicine
		2. Indigenous perception of time (C) + reduced reciprocal respect (M) ➔ decreased trust (O_i_) ➔ reduced likelihood to engage
	Indigenous health beliefs and spirituality	1. Strong self‐alignment with an indigenous world view (C) + perception of world view acceptance (M) ➔ increased trust (O_i_)
		1a. Increased trust (C) + decreased anxiety (M) ➔ increased engagement (O_f_)
	Holistic learning style	1. HCP has low communication skills (C)+ decreased perception of world view acceptance (M) ➔ increased anxiety (O_i_)
		1a. Decreased perception of world view acceptance (C) + increased anxiety (M) ➔ decreased trust (O)
	Importance of community	1. High alignment with indigenous world view on the importance of family (C) + inclusion of family and community in the decision‐making process (M) ➔ increased trust (O_i_)
		1a. Increased trust (C) + decreased anxiety (M) ➔ increased engagement in SDM (O_f_)
Additional contexts (C_3_, C_4_)	Language barriers	1. High alignment with indigenous world view (C) + strong HCP communication skills displayed through appropriate response to indigenous language style (M) ➔ increased ability to engage in the SDM process (O)
	System and institutional support	1. Historical trauma (C) + lack of system support (M) ➔ decreased trust (O)
		2. Historical trauma (C) + system support offered (M) ➔ increased trust (O_i_) + decreased anxiety (O_i_)
		2a. System support (C) + increased trust (M) + decreased anxiety (M) ➔ increased engagement (O_f_)
Reciprocal respect *Mechanism set* (M_1_)	HCP cultural sensitivity and awareness	1. Unidentified context (C) + culturally sensitive and aware HCP (M) ➔ decreased anxiety (O_i_)
		1a. Culturally sensitive HCP (C) + decreased anxiety (M) ➔ increased trust (O_F_)
	HCP communication	1. Unidentified context (C) + strong communication skills displayed through an appropriate understanding of communication cues (M) ➔ increased trust (O_i_)
		1a. Increased trust (C) + decreased anxiety (M) ➔ patient engagement in SDM (O_f_)
	Power balance	1. Historical discrimination (C) + ongoing power imbalance (M) ➔ reduced ability to engage in SDM (O)
	HCP relationship and advocacy	1. Unidentified context (C) + lack of reciprocal respect (M) ➔ inability to trust HCP (O_i_)
		1a. Lack of trust with HCP (C) + increased anxiety (M) ➔ decreased engagement in SDM (O_F_)
		2. Unidentified context (C) + patient perception of reciprocal respect (M) ➔ increased trust (O_i_)
		2a. Trust with HCP (C) + decreased anxiety (O_i_) ➔ patient engagement in SDM (O_F_)
Perception of world view acceptance *Mechanism set* (M_2_)	Acceptance of ceremony and spirituality	1. High alignment with an indigenous world view (C) + high HCP respect for indigenous medicine (M) ➔ increased trust (O_i_)
		1a. HCP respects indigenous medicine (C) + increased trust (M) ➔ decreased anxiety (O_F_)
		2. High alignment with an indigenous world view (C) + HCP incorporation of patient beliefs toward health (M) ➔ increased patient trust (O)
	Acceptance of family and community	1. High alignment with an indigenous world view (C) + HCP demonstration of willingness to include family (such as the extension of consultation time) (M) ➔ Perception of world view acceptance (O_i_)
		1a. HCP includes the patient's family (C) + perception of world view acceptance (M) ➔ increased level of reciprocal respect (O_i_)
		1b. Perception of world view acceptance (C) + increased level of reciprocal respect (M) ➔ increased trust with HCP (O_F_)
Culturally appropriate knowledge translation *Mechanism set* (M_3_)	Storytelling as education	1. High alignment with indigenous world view (C) + successful use of storytelling as information exchange between patient and HCP (M) ➔ increased perception of world view acceptance (O_i_)
		1a. Increased perception of world view acceptance (C) + *increased comprehension* (M)➔ increased trust (O_f_)

Abbreviations: CMO, context‐mechanism‐outcome; HCP, health‐care provider; SDM, shared decision making.

In the analysis, we also used intermediate outcomes (_I_O): outcomes of interactions between C and M, but preceding the final O. All CM _I_OOs were identified based on (a) impact on trust and/or world view and (b) apparent impact on decision making. In some CMOs, trust and anxiety also functioned as mechanisms, while world view could be both context and mechanism. The description of these contexts, the components of mechanisms, and their influence on outcomes are outlined in the following section.

#### 3.2.1 Macro‐context and SDM

In our review, seven documents highlighted cultural discrimination and historical abuse as a macro‐context, which has the potential to influence indigenous SDM in the health‐care system. Assimilation policies and discrimination continue to influence the health‐care system.^40^, ^41^, ^42^ This discrimination causes discord and alienation^43^, ^44^ and a pressure for indigenous patients to conform to a Western world view.^3^, ^36^ Historical abuse and cultural discrimination impact all mechanisms in our revised programme theory, particularly perception of world view acceptance, power balance within reciprocal respect, trust, and anxiety. This macro‐context dissuades indigenous patient engagement in SDM.

#### 3.2.2 Contexts in relation to mechanisms and outcomes

Demographics (C_1_) such as gender, age, and location provided a context for the subsequent context of indigenous world view. Colonialism altered gender roles in most indigenous societies. As a result, in some, but certainly not all, indigenous communities' men became the decision makers within each household and women were stripped of this right.^45^, ^46^ Older individuals are expected to care for the younger generations^47^ and are seen as knowledge keepers in the community.^48^ Younger people could be less likely to adhere to an indigenous world view than older people.^49^, ^50^ Those who live on‐reserve are more likely to have a stronger tie to indigenous beliefs^51^, ^52^ than are those who live within urban centres who are more likely to align with Western world views.^51^ Location was also an indicator of access to health care because of cost and distance.^50^, ^53^, ^54^

Indigenous world view is both a context (C_2_) and a mechanism (M_2_) in our revised programme theory. Indigenous world view as context is the degree to which patients adhere to indigenous world view, and patients' perception of how much their indigenous world view is accepted by the practitioner acts as a mechanism. While indigenous individuals differ in the degree to which they adopt a traditional world view,^37^, ^51^ indigenous world view is complex and composed of perception of time, health beliefs and spirituality, holistic learning style, and importance of community. We discuss these components of indigenous world view and how they relate to particular mechanisms within the programme theory in the following paragraphs.

People with indigenous world view may perceive time as relational and non‐linear,^45^, ^55^, ^56^, ^57^ which could in part contribute to patients not meeting appointments or following pharmaceutical schedules. Health‐care providers may misinterpret this as a lack of patient compliance.^55^, ^58^ However, patient noncompliance can be the result of other social and geographic conditions. This aspect of world view context may impact the mechanism of reciprocal respect, thereby decreasing trust and subsequent SDM engagement.

Indigenous health beliefs incorporate not only the physiological but also psychological, cultural, and spiritual elements of existence. 37, 42, 59, 60, 61 Indigenous spirituality acknowledges the unseen forces with which humans have a relationship: Everything is connected to the web of life.^45^ To heal, some indigenous patients use ceremony, herbs, and traditional healers, with emphasis on unifying the mind and body.^62^ This includes having a healthy and reciprocal relationship with the land. Because this part of the context of indigenous world view does not align with Western medicine conceptions of health, the context could result in decreased perception of world view acceptance and less trust and engagement in SDM.

Indigenous world view involves holistic learning style where learning and gaining knowledge are inseparable from living; indigenous world views treat knowledge acquisition as a personal, multigenerational, and communal transformation.^37^, ^59^ When this component of indigenous world view is a contextual factor, the mechanism of culturally appropriate knowledge translation is present and triggers an outcome of increased trust and decreased anxiety which in turn results in SDM engagement.

Indigenous culture places an emphasis on harmonious relationships between the individual and his or her community.^37^, ^56^, ^59^ Community is important because the individual's identity and spirituality are influenced by reciprocal connection to the social resources of the community.^36^, ^58^ Raising children involves more individuals than the immediate family and close relatives but extends to community members who may not be related.^37^, ^63^, ^64^, ^65^, ^66^ If these relationships are perceived to be unimportant in the health care interaction, the patient's world view is both not accepted and disrespected. The likelihood the patient will trust the practitioner and engage in SDM is diminished.

The final two contexts that impact the mechanisms in the programme theory are system and institutional support (C_3_) and language barriers (C_4_). Western biomedicine typically defines most health care interactions. Part of respecting traditional practices means accepting the holistic elements of the culture and recognizing that ceremony, values, place, and location cannot be separated. It also means showing a willingness to acknowledge different ideas about disease causality and symptomatology.^38^, ^67^ When the system does not promote reciprocal respect through the promotion of HCP cultural awareness, communication, relationships, and advocacy, trust decreases and anxiety increases. Shared decision making does not occur. In this context, the power imbalance between the practitioner and patient is exacerbated, leading to similar consequences.

Language barriers are another context that can lead to a decreased perception of world view acceptance and subsequent decreased trust and increased anxiety. Indigenous communication is highly contextual and relies on nonlexical aspects.^68^, ^69^ For example, this process of communication includes the expression of individual characteristics such as patience and humility, which are communicated through how a person walks, sits, and talks.^64^ Further, foundational linguistic differences exist between Western and indigenous languages; certain medical terms in some indigenous languages cannot be translated because an equivalent may not exist.^38^, ^60^, ^62^, ^68^

#### 3.2.3 Descriptions of mechanisms

**Figure 3:**
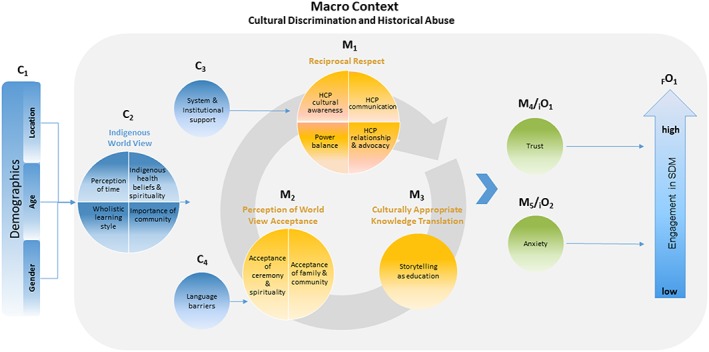
Revised programme theory for indigenous patients

In our revised programme theory (Figure 3), the mechanism of reciprocal respect (M_1_) has four components: HCP cultural awareness, HCP communication, power balance, and HCP relationship and advocacy. Cultural awareness is characterized by an HCP's consideration of indigenous world view. This includes recognizing that family structures, the connection to land and community, traditional healing, and spirituality are often important facets of indigenous life. 36, 42, 43, 61, 70 Effective communication is enabled when the beliefs, values, and language of the indigenous person are incorporated into the interaction.^63^ Health‐care providers without effective communication skills may erode the patient's perception of cultural respect and pride.^58^, ^71^ Health‐care providers must work to reduce power imbalances in order to build reciprocal respect reduce patient anxiety and increase trust.^4^ When an individual perceives there is a more balanced relationship with the HCP, trust increases and anxiety decreases,^41^ making SDM engagement more likely.^5^, ^43^

Advocacy involves including the indigenous patient in the decision‐making process in a substantive way.^49^ Health‐care providers can foster inclusivity by inviting the patient to take an active role in the process^20^, ^40^, ^43^, ^56^, ^72^, ^73^, ^74^ and display a genuine interest in their patient by making a greater effort to communicate with them.^38^, ^40^, ^74^, ^75^ Continuity of care from the same HCP is also important, allowing trust to establish and continue.^63^, ^72^ When the patient has this type of relationship with the HCP, trust is higher and anxiety is lower.

The second collection of mechanisms in our revised programme theory is the patient's perception of world view acceptance (M_2_). This entails two components: acceptance of ceremony and spirituality and acceptance of family and community. Demonstrating respect for indigenous ways of knowing, communication, and spirituality are important to fostering a perception of world view acceptance,^39^, ^41^, ^67^, ^76^ which can be achieved by incorporating traditional medicine into care.^13^, ^53^, ^77^, ^78^ Acceptance of family and community when the HCP demonstrates a willingness to make the proper arrangements and resources (including time) for the involvement of family and community. The result is patients feel their world view is more accepted.^64^, ^65^, ^74^, ^79^ In this situation, the outcome of SDM engagement would be expected.

When health information is translated using culturally appropriate knowledge (M_3_), an indigenous patient will be more likely to engage in SDM and have greater trust in the practitioner and less anxiety. An essential form of culturally appropriate knowledge translation is storytelling. Storytelling passes information through personalized communication and has been used for generations.^37^, ^48^, ^71^ It is a means of transmitting information, including health information that incorporates cultural and spiritual values. Indigenous world view promotes learning through observation.^55^ Health‐care providers must be aware of this specific form of learning and recognize that silence does not equate to disengagement. When this aspect of world view is accepted, there is increased comprehension among patients and thereby increased trust.

#### 3.2.4 Revised programme theory

Figure 3 outlines the revised programme theory and illustrates all components of contexts and mechanisms. Although elements of our revised programme theory consider HCP mechanisms, Figure 3 focuses on the indigenous patient's side of the consultation process. Beginning with the context of demographics, followed by the context of indigenous world view, this category incorporates the macro‐context of discrimination and historical abuse. This macro‐context overlays the remainder of the programme theory. The three remaining categories of mechanisms (reciprocal respect, perception of world view acceptance, and culturally appropriate knowledge translation) and their respective attributes are within the iterative circle. This represents the cyclical, interconnected nature of indigenous beliefs. System and institutional support provide context for the mechanism of reciprocal respect, and language barriers are the context for the perception of world view acceptance mechanism. The four contexts and three mechanisms interact to generate the intermediate outcomes of trust and anxiety. How trust and anxiety manifest will determine the level a patient is able to engage in the SDM process (final outcome). As a mechanism, greater trust may result in more SDM engagement, while the mechanism of greater anxiety may lead to less engagement in SDM.

Shared decisions for indigenous patients can entail choosing Western‐based treatments. Our revised programme theory predicts that younger patients with lower alignment to indigenous world view are more likely to choose Western treatments. Contexts and mechanisms that decrease trust are more likely to dissuade an SDM process regardless of the patient's treatment path.

Compared with the initial programme theory, the indigenous patient programme theory relies heavily on trust and anxiety for SDM engagement to occur. These intermediate outcomes that are inspired by reciprocal respect, perception of world view acceptance, and culturally appropriate knowledge translation become the generative forces for SDM engagement. The contextual conditions for indigenous patients are complex.

## 4 DISCUSSION

The focus of this realist synthesis was to revise our previous programme theory to understand and predict how trust and world view impact engagement in SDM for indigenous patients. As part of indigenous patients' experiences as colonialized people, their levels of trust with institutions have been negatively impacted. They tend to have a world view that is distinct from that of nonindigenous people. Our revised programme theory depicts the complex and interconnected manner in which a combination of trust (as intermediate outcome and mechanism) and world view (as contexts and mechanisms) allows for (or do not allow for) engagement in SDM. Our synthesis revealed that for indigenous patients, interactions with HCPs are overlaid by a macro‐context of cultural discrimination and historical abuse. Other contexts that impact mechanisms included indigenous world view, system and institutional support, and language barriers. The mechanisms of reciprocal respect, perception of world view acceptance, and culturally appropriate knowledge translation had an effect on patients' trust and anxiety. These intermediate outcomes could then act as mechanisms to generate levels of SDM engagement.

Appreciating and attending to indigenous world views may allow Canadian HCPs to help patients to have better experiences with the health‐care system. Castellano interprets indigenous world views as the roots of a tree and the earth as the spiritual world.^80^ The leaves represent individual behaviours; small branches represent protocols and community customs; large branches represent the rules governing relationships; and the trunk represents the ethical values of good and evil. She adds that the “world is alive, conscious, and flowing with knowledge and energy.” Indigenous world views are also relational. People and other things (spiritual, individuality and community) come together to help each other.^36^ Despite their similarities, each indigenous society has its own world view, values, and ethics. Generally, they do not follow one single philosophy, belief, or moral code.^80^ Although we have examined world view in this realist synthesis, its complexities and how it relates to SDM warrant further investigation.

### 4.1 Practical implications

Some indigenous people consider diseases such as cancer to be the result of colonial contact, and consequently, such colonial illnesses carry a stigma within the communities and a distrust of Western medicine.^52^, ^81^ Shared decision making may bridge the gap between stigma and reality; indigenous world views are likely essential to reach SDM. Some indigenous patients may feel their beliefs are more in line with Western than indigenous world views, or they may believe aspects of both.^82^ Understanding that indigenous patients may hold different world views may help HCPs engage in conversations about a patient's world view. These conversations may customize the consultation to the patient's needs and values and promote SDM.

While this study is the first to provide a programme theory on indigenous trust and world view and their impact on SDM, previous studies have discussed similar barriers. In a systematic review of barriers to health care access, one study found that rural location and communication differences can impede an indigenous patient's ability to access health care.^70^ This review encouraged greater understanding of interventions to increase access and utilization among indigenous populations. Another review of chronic disease interventions in primary health care acknowledged the flexibility of enablers and barriers, the contingencies on which interventions are effective, and the interrelatedness of enablers and barriers.^83^ Similar to our findings, the authors of this review recognized the necessity of evidence base to successfully design, implement, and sustain interventions for indigenous people. Our revised programme theory extends the results of these systematic reviews to identify more barriers and enablers to SDM and provides theoretical connections between them.

The revised programme theory may assist in building trust and improving inter‐cultural understanding within the health‐care system. Health‐care providers can foster reciprocal respect and accept the patient's world view through the incorporation of traditional medicine practices. Building on existing cultural safety initiatives,^84^, ^85^, ^86^ this revised programme theory will provide a foundation for HCPs to meet culturally specific needs of indigenous patients.

Overcoming historical, institutional, and systematic barriers requires changes at a policy level. Cultural and institutional barriers could be addressed by consulting and engaging indigenous leadership at the community level. Adopting the Truth and Reconciliation Commission of Canada health recommendations and designing a plan of action will improve the health of indigenous people. Culturally appropriate supports that are patient focused and use the patient's first language should be implemented. Examples of supports can include incorporating community‐level resources in care,^3^, ^48^ offering support to families particularly in instances where homecare is used,^87^ and informing patients of different resource options, including information resources^75^, ^79^ in the patient's first language.^88^ Moreover, institutions should strive to uphold a patient's right to incorporate their own health beliefs and treatment options into their decision‐making process. Using our revised programme theory, policymakers can identify areas where system‐level improvements can be made and can implement change. The incorporation of indigenous HCPs or peer navigators into Western health systems may guide and support decision‐making processes.

### 4.2 Limitations

This research used a limited number of databases, which may introduce a selection bias. The inclusion of iPortal helps negate this bias as it allowed us to use a variety of diverse sources. Also, our analysis may be limited as it was conducted by mostly nonindigenous researchers and without stakeholder input. While our search strategy was developed in collaboration with an indigenous scholar, our analysis may be biased because of our own world views. Because we began this review by using a nonindigenous informed programme theory, this may affect the reproducibility of the results. However, by testing the theory with indigenous patients, the theory can be confirmed, refuted, or refined to reflect engagement in SDM by indigenous patients.

Throughout this realist synthesis, we extracted data that reflected the difficulty in translation between indigenous language and Western comprehension of terms. We recognize that indigenous spirituality is unique and cannot be encapsulated by nonindigenous language or explained in categories. Nevertheless, we believe the revised programme theory would be strengthened through testing with indigenous patients.

We recommend testing this programme theory with indigenous patients to ensure it accurately represents indigenous patients' experiences with SDM. Testing will allow confirmation, refinement, or refutation of the proposed programme theory, with the overall aim of implementing the findings. As there is preliminary evidence that access and treatment play a role in indigenous patients' trust and world views, tailoring the programme theory for trust and world view to specific indigenous patients would strengthen the potential impact of this programme theory.^81^

### 4.3 Conclusion

This realist synthesis examined the contexts, mechanisms, and outcomes relevant to indigenous patients' engagement in SDM. Fostering indigenous world view includes culturally appropriate services to mitigate the negative impact of the macro‐context, cultural discrimination, and systemic and historical abuse. Our revised programme theory postulates that when HCPs accept and include indigenous world views into the decision‐making process, trust is facilitated, and patient anxiety is reduced. Indigenous patient engagement in SDM may rely on HCP and health‐care system ability to integrate multiple world views. This realist synthesis provides clinicians and policymakers a deeper understanding of the complex configurations that influence indigenous patient engagement in SDM and offers possible avenues for improvement. Future tests of the programme theory will offer options for refinement and application in various health settings.

## CONFLICT OF INTEREST

The authors declare no conflict of interest.
